# *Borrelia miyamotoi in vivo* antigenic variation demonstrated by serotype reisolations from infected mice

**DOI:** 10.1128/iai.00484-24

**Published:** 2025-03-07

**Authors:** Brittany A. Armstrong, Kevin S. Brandt, Robert D. Gilmore

**Affiliations:** 1 Bacterial Diseases Branch, Division of Vector Borne Diseases, National Center for Emerging and Zoonotic Infectious Diseases, Centers for Disease Control and Prevention 164543 , Fort Collins, Colorado, USA; Washington State University, Pullman, Washington, USA

**Keywords:** *Borrelia miyamotoi*, relapsing fever, antigenic variation

## Abstract

Relapsing fever *Borrelia* (RFB) employs antigenic variation to alter its surface protein structure in response to host immune pressure. This process occurs by the single translocation of archived variable major protein (Vmp) pseudogenes into a *vmp* expression locus. *Borrelia miyamotoi*, phylogenetically grouped with RFB, has the genetic makeup for antigenic variation, but it has not been determined whether *B. miyamotoi* can create new variant serotypes *in vivo*. We inoculated mice with a non-clonal parental *B. miyamotoi* CT13-2396 strain with a known Vmp majority serotype with spirochete isolation at various days post-infection. The *vmp* that determined the reisolated variant serotype was identified by PCR of the expression locus followed by DNA sequencing of the amplified product. For each mouse reisolate, new variants replaced the parent majority serotype. Moreover, some mice produced additional variant reisolates days apart, indicative of the presentation seen in relapsing fever infections. Infection of mice with a clonal population resulted in the elimination of the inoculated serotype and isolation of new variants. Mouse serum obtained following infection revealed IgM antibodies reactive to the parent Vmp serotype, suggesting that the immune response eliminated or greatly reduced the majority population. These results demonstrated that *B. miyamotoi* reisolated from infected mice exhibited serotype populations differing from the inoculated strain, indicating the spirochetes underwent antigenic variation to evade the host’s immune response. However, whether the observed variation occurred by way of outgrowth of minority populations or by translocation of archived pseudogenes to the expression locus creating new variants awaits further study.

## INTRODUCTION

*Borrelia miyamotoi* is an emerging tick-borne bacterial pathogen of humans. The spirochete was first identified from ticks in Japan and was later recognized to cause human disease in Russia in 2011 and from 2013 to the present in patients throughout Japan, Europe, and North America (1–8). Although phylogenetically grouped with relapsing fever *Borrelia* (RFB), *B. miyamotoi* is not transmitted by *Ornithodoros* spp. (soft ticks) but rather *Ixodes* spp. (hard ticks), leading to the designation of the disease as hard tick relapsing fever (HTRF), also referred to as *Borrelia miyamotoi* disease and hard tick-borne relapsing fever (8–12). HTRF case definition lists fever, myalgia, arthalgia, and headaches as common manifestations with relapsing fevers less frequently documented (13–15). *Borrelia burgdorferi*, the causative agent of Lyme disease, is also transmitted by *Ixodes* spp. (ticks). Therefore, the geographic regions for HTRF and Lyme disease cases overlap, and accurate confirmatory diagnoses can be challenging.

Relapsing fever is caused by several species of arthropod-borne spirochetes, the more extensively studied being New World species, *Borrelia hermsii* and *Borrelia turicatae*, found in North America, transmitted by soft ticks *Ornithodoros hermsii* and *Ornithodoros turicata*, respectively (16–18). Clinically, infection with these pathogens follows a course of recurring fevers interspaced with non-febrile periods. The ability of infecting borreliae to alter their surface protein structure to evade the host’s adaptive immune response results in serotype variation, allowing spirochete proliferation and a relapse of fever (19, 20).

RFB serotypes are determined by the synthesis of a single variable major protein (Vmp) localized on the cell surface. Vmps are divided into two groups, variable small proteins (Vsps) and variable large proteins (Vlps), that are approximately 20 and 36 kDa, respectively (21, 22). Vmps are encoded by transcriptionally silent pseudogenes localized on multiple linear plasmids (lps), and *vmp* expression occurs at a single locus driven by an active promoter (23–25). During host infection, RFB antigenically switches to a new serotype to evade the adaptive immune response by replacing one *vmp* with another via gene conversion at the expression locus (26–28). In *B. hermsii*, this translocation occurs by intragenic recombination and can involve chimeric representation of separate Vmp-encoding genes (29, 30). While infectious RFB may consist of a predominant serotype population, minority populations may arise pre-infection by spontaneous recombination, allowing for opportunistic growth in the host as a mechanism to survive antibody killing pressure (26, 29).

Genome sequencing of multiple strains available in public databases, including a recent comprehensive analysis of geographic strain diversity, has shown that *B. miyamotoi* possesses a repertoire of transcriptionally inactive Vmp-encoding genes on multiple plasmids similar to other RFBs that indicated this spirochete is capable of antigenic variation (31–33). These reports have identified a single *B. miyamotoi* expression locus with an active promoter for *vmp* transcription enabling one *vmp* to be expressed similar to other RFBs (32). The first study to demonstrate *B. miyamotoi* antigenic variation was performed by Wagemakers et al., who found that serotype switching took place when borreliae were subjected to monospecific antibody pressure *in vitro* (34). Our laboratory expanded on that finding with a study showing polyclonal antibody killing of a *B. miyamotoi* parent strain resulted in growth of new antibody resistant variants (35). Both studies were performed *in vitro*, providing evidence that *B. miyamotoi* antigenically switched under antibody pressure, but antigenic variation *in vivo*, i.e., during mammalian infection, has not been demonstrated. We hypothesized that antigenic variation could occur during mammalian infection in a similar fashion as demonstrated *in vitro* in response to natural adaptive immunity. The goals of this study were to (i) isolate and cultivate *B. miyamotoi* from mouse blood post-inoculation and (ii) determine whether antigenic variation occurred by determining the serotype of blood reisolates. We report successful *B. miyamotoi* reisolation from mouse blood following needle inoculation infection and the identification of new variant serotypes that replaced the parent serotype.

## RESULTS

### Stock parent strain CT13-2396 determined non-clonal

The strain CT13-2396 genome sequence in GenBank (https://www.ncbi.nlm.nih.gov/datasets/genome/GCF_001767415.1/) indicated that the Vmp-encoding gene in the expression locus on lp41 was AXH25_RS05645 (old locus AXH25_04655), annotated as Vlp5, and therefore represented the majority serotype strain used for sequencing by Kingry et al. (31) and provided to our laboratory. Strain LB-2001 used in previous reports was serotype Vsp1 (34, 36); therefore, it is notable that these two North American strains differed in their Vmp-encoding gene in the expression locus. For simplicity, Vmp gene AXH25_RS05645 (Vlp5) and the corresponding protein will be referred to as VlpA throughout the text, with other Vlp proteins and genes referenced in this report also designated by a letter. A legend corresponding to the letter designations with the GenBank gene locus number is shown in Table 1.

**TABLE 1:** Information legend of Vmps described in Experiments 1–4

Vmp designation	Gene	Experiment reisolate^*a*^	Subfamily	Plasmid	Duplicate gene/plasmid
VlpA	AXH25_RS05645	1	Gamma	lp41	RS05360/lp26
VlpB	AXH25_RS05650	1, 2, 3, and 4	Delta	lp41	RS05365/lp26
VlpC	AXH25_RS05655	1, 2, 3, and 4	Delta	lp41	
VlpD	AXH25_RS05665	1, 2, 3, and 4	Delta	lp41	RS05380/lp26
VlpE	AXH25_RS05670	1, 2, 3, and 4	Delta	lp41	RS05385/lp26
VlpF	AXH25_RS05680	1, 2, 3, and 4	Delta	lp41	
VlpG	AXH25_RS05355	2, 3, and 4	Delta	lp26	
VlpH	AXH25_RS05370	1, 2, 3, and 4	Delta	lp26	
VlpI	AXH25_RS05465	1, 2, 3, and 4	Delta	lp30	
VlpJ	AXH25_RS05480	3	Alpha	lp30	
VlpK	AXH25_RS05195	1, 2, 3, and 4	Delta	lp20-2	
VlpL	AXH25_RS05285	1, 2, 3, and 4	Delta	lp23	
Vsp	AXH25_RS05675	3 and 4		lp41	RS05390/lp26

^*a*^ Experiments with reisolates containing reads to the indicated Vmp.

**Fig 1:**
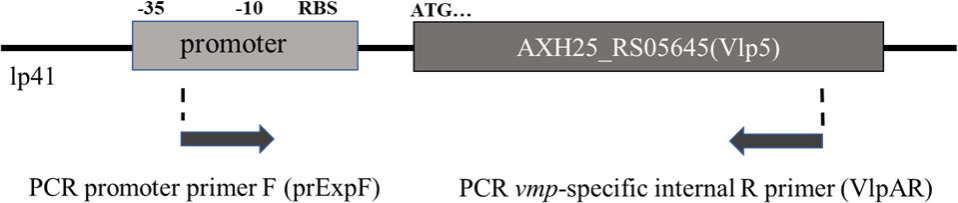
Expression locus PCR analysis. Illustration of PCR identifying *vmp* contiguous with the active promoter. The example shows the VlpA gene designated by GenBank reference number AXH25_RS05645 annotated as Vlp5. Arrows designate the forward primer in the promoter region (prExpF), with the reverse primer located internal to *vmp* (VlpAR in this example). The expression locus plasmid location is the 41 kb linear plasmid (lp41). The prokaryotic promoter regions –35 and –10, the ribosome-binding site (RBS), and the ATG codon start site for the expressed gene are designated.

We confirmed by PCR that VlpA was in the expression locus of our low-passage stock CT13-2396 using a forward primer specific for a region within the promoter (prExpF) and an internal reverse primer, VlpAR, specific only for VlpA (Table 2; Fig. 1). The 842 bp PCR fragment was determined by Sanger sequencing to be *vlpA*. Further PCR analysis of the expression locus of the stock strain, using Vlp internal reverse primer Vlp25R, produced a less prominent amplicon. Subsequent Sanger DNA sequencing of the amplicon was unreadable due to overlapping peaks in the chromatogram, in contrast to the sequencing performed with the VlpA amplicon. This result suggested that the Vlp25R amplified multiple PCR products all approximately the same size, representing additional variant serotypes in the stock strain. Therefore, PacBio sequencing was performed on the Vlp25R PCR product, generating reads that created a profile revealing additional expression loci *vlp*s (Table 3). The read data provided insight into the serotype representations within the stock strain population ranging from the highest, VlpB with >15,000 reads, to VlpJ and others with 1 read (Table 3). Two archived genes, AXH25_RS05350 and AXH25_RS05505, were not represented. VlpA is represented in the read data with less than 5,000 reads, which at first glance contradicts that this serotype predominates in the parent. We attribute this observation to the use of the Vlp25R primer, which was designed to amplify several *vlp* pseudogenes, even those that had some internal base mismatches, thus entailing the 50°C annealing temperature during PCR. Indeed, an alignment of the Vlp25R primer with the corresponding regions of CT13-2396 Vlp subfamily gamma genes showed that there are eight mismatches with VlpA (Fig. S1). Therefore, we postulate that *vlpA* was poorly amplified with Vlp25R, resulting in a reduced number of reads by PacBio sequencing. This scenario can also explain the low read numbers of gamma subfamily genes *vlpG* and *vlpJ*. The genes with the most reads belong to the delta subfamily (Fig. S1).

**TABLE 2:** Primer sequences for expression locus PCR and recombinant protein cloning

Primer	Sequence	Target and purpose
prExpF	ATAAAGAATTTGAAAAGTAAGATTCTTGCAC	Expression locus promoter for PCR
VlpAR	CACCAGCATTCTGAGAAGTAGCTAATTTGGCA	*vlp5* (AXH25_RS05645); also specific for *vlpC2*, strain LB-2001; reverse primer for PCR
Vlp25R	CCACTATCAACTGTATTTCTTATTGCTA	Conserved internal region of 25 *vlp* beta, delta, and gamma subfamilies; reverse primer for PCR
Vlp4R	CTTGCTTCATCTATTTTTTCTTTTGC	Conserved internal region of four *vlp* alpha subfamilies, reverse primer for PCR
Vsp1R	GCACTTTTTGCATGAGCATC	Conserved internal region of *vsp* family, reverse primer for PCR
VlpB-F	CATCATCACCACCATCACGGAGGGGAAGATCCACAA AAG	Forward and reverse primers to amplify *vlpB* for cloning in expression plasmid, underlined nucleotides designed for pETite N-His vector
VlpB-R	GTGGCGGCCGCTCTATTAGTTTTGTACACTAGTTGTTGCTTCTGC	
VlpC-F	CATCATCACCACCATCACGGAGGGGAAGATCCACAAAAG	Forward and reverse primers to amplify *vlpC* for cloning in expression plasmid, underlined nucleotides designed for pETite N-His vector
VlpC-R	GTGGCGGCCGCTCTATTAGTTTTGTACACTAGCTGTTACTTC	
VlpD-F	CATCATCACCACCATCACGGAGGGGAAGATCCACAAAAG	Forward and reverse primers to amplify *vlpC* for cloning in expression plasmid, underlined nucleotides designed for pETite N-His vector
VlpD-R	GTGGCGGCCGCTCTATTAGTTTTGTGCACTAGCTGTTACTTC	
Vsp1-F	GAAGGAGATATACATATGTGTGGAAGTGGGGGACCGGCA	Forward and reverse primers to amplify *vsp1* for cloning in expression plasmid, underlined nucleotides designed for pETite C-His vector
Vsp1-R	GTGATGGTGGTGATGATGTGAAGATTGACCAGCAGTTAA	

**TABLE 3:** CT13-2396 subdominant Vlp populations from parent freezer stock: PacBio sequencing results of PCR product of expression locus with Vlp25R primer^*a*^

Vlp locus and figure designation	Reads
AXH25_RS05650 **VlpB**	15,615
AXH25_RS05670 **VlpE**	14,224
AXH25_RS05655 **VlpC**	6,228
AXH25_RS05370 **VlpH**	6,212
AXH25_RS05645 **VlpA**	4,816
AXH25_RS05265	3,604
AXH25_RS05465 **VlpI**	2,322
AXH25_RS05665 **VlpD**	1,296
AXH25_RS05510	424
AXH25_RS05490	327
AXH25_RS05500	324
AXH25_RS05195 **VlpK**	176
AXH25_RS05495	137
AXH25_RS05270	83
AXH25_RS05285 **VlpL**	41
AXH25_RS05260	32
AXH25_RS05250	18
AXH25_RS05680 **VlpF**	17
AXH25_RS05255	7
AXH25_RS05355 **VlpG**	6
AXH25_RS05375	6
AXH25_RS05395	6
AXH25_RS05485	3
AXH25_RS05190	2
AXH25_RS05475	2
AXH25_RS05660	2
AXH25_RS05685	2
AXH25_RS05085	1
AXH25_RS05280	1
AXH25_RS05290	1
AXH25_RS05480 **VlpJ**	1
AXH25_RS05350	0
AXH25_RS05505	0

^*a*^ Duplicate genes were reflected by identical number of reads.

The data suggest that multiple archived genes were represented in the stock strain with some at very low frequency compared to others. A faint amplicon was also detected using the Vsp1R primer, indicating a Vsp serotype was also represented. No amplicons were observed with the Vlp4R primer. Overall, these results indicated that the parent CT13-2396 stock strain used for the animal experiments was non-clonal and suggested a majority serotype of VlpA when also considering the annotated CT13-2396 GenBank sequence for the expression locus.

### Vmp variation during mouse infection

**Fig 2:**
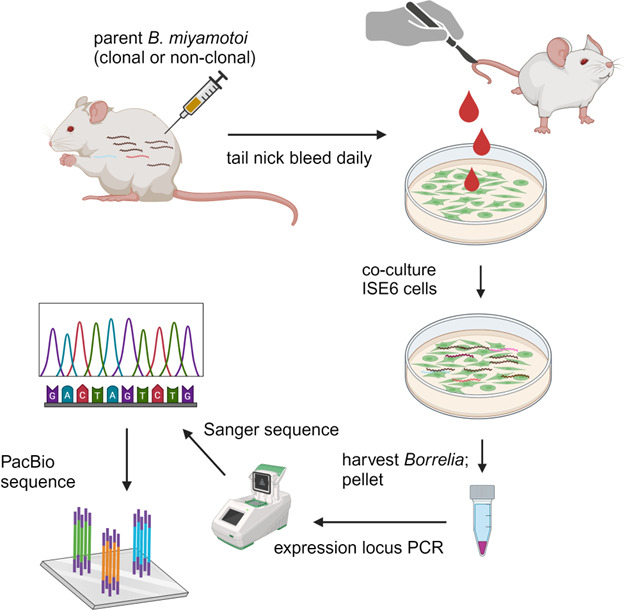
Experimental design for mouse *B. miyamotoi* inoculations, reisolation of *B. miyamotoi* from blood, culture expansion, and expression locus PCR for DNA sequencing.

In our experimental design, we injected the parent low-passage non-clonal strain into mice and collected blood during the infection from which *B. miyamotoi* was isolated by co-cultivation with ISE6 tick cells (Fig. 2). Reisolates were subjected to expression locus PCR with primer pairs consisting of prExpF and each of VlpAR, Vlp25R, Vlp4R, and Vsp1R. Resultant PCR products were gel purified and Sanger sequenced to identify the archived gene in the expression site to determine the serotype. When Sanger sequencing showed an unreadable profile of overlapping peaks, what appeared to be a single amplicon band on a gel actually consisted of multiple amplicons with no discernible size differences resolvable by agarose electrophoresis. Therefore, PacBio sequencing was performed to identify the *vmps* represented in the mixed amplicon sample (Fig. 2). Four experiments were performed.

### Experiment 1: serotypes emerge following isolation from mice infected with the uncloned parent strain

**Fig 3:**
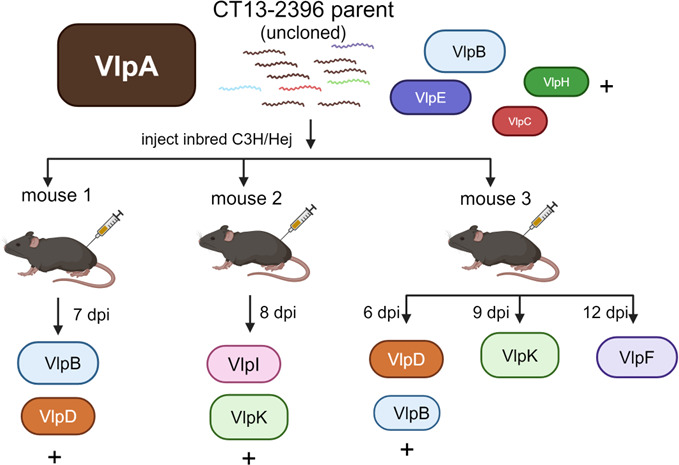
Vlp serotype determinations from Experiment 1 mouse reisolates. Strain CT13-2396 parent strain inoculated into C3H/Hej mice (*n* = 3). Uncloned parent strain is illustrated as VlpA serotype majority with subdominant serotypes (B, E, H, and J) listed according to reads determined by PacBio sequencing in Table 4. Vlp genes identified as being in the expression locus are indicated by letters and are color coded with corresponding GenBank locus reference tag designations listed in Table 3. (+) indicates additional subdominant serotypes that may be present in the populations. Sequencing reads for Mouse 1 (7 dpi), Mouse 2 (8 dpi), and Mouse 3 (6 dpi) reisolates with the legend for gene locus tag designations corresponding to the letters are listed in Table S1.

We inoculated inbred C3H/Hej mice (*n* = 3) with strain CT13-2396 non-clonal parent and isolated *B. miyamotoi* from the blood of each mouse at various days post-infection (dpi) (Fig. 3). Expression locus PCR of reisolates from each mouse using the prExpF/VlpAR primer set failed to produce a VlpA amplicon, indicating this serotype had been diminished below detection or was eliminated during the infection. However, expression locus PCR with the prExpF/Vlp25R primer set generated an amplicon from each mouse reisolate. Mouse 1 reisolate at 7 dpi consisted of multiple variants (VlpB and VlpD with the highest number of PacBio reads; Fig. 3; Table S1). Mouse 2 reisolate at 8 dpi also had multiple variants (VlpI and VlpK with most PacBio reads; Fig. 3; Table S1). Mouse 3 reisolate consisted of multiple variants at 6 dpi (VlpD and VlpB with most PacBio reads; Fig. 3; Table S1), but the reisolate at 9 dpi indicated a single variant VlpK, and a reisolate at 12 dpi indicated a single variant VlpF as determined by clean Sanger sequencing peak profiles (Fig. 3). No PCR products were generated using the prExpF/Vlp4R or prExpF/Vsp1 primer sets.

**TABLE 4:** *B. miyamotoi* mouse infections for blood reisolation experiments

Experiment	Inoculation isolate	Pre-inoculation growth medium	Passage number	Number of mice	Bleed schedule	Days tail nicked	Terminal bleed (DPI)
1	Parent isolate	BSK-R^*a*^	4	3	Staggered	15	42
2	C3H/HeJ isolate	BSK-R	0	3	Staggered	15	42
3	CD-1 isolate	ISE6 co-culture	1	3	Daily	15	15
4	Clonal isolate	ISE6 co-culture	7	3	Daily	15	15

^*a*^ BSK-R, Barbour-Stoenner-Kelly medium.

This experiment showed that (i) parent serotype VlpA was diminished below detection or eliminated from all mice post-infection; (ii) reisolated serotype variants differed to some extent between mice despite being inoculated with the same parent strain; and (iii) reisolates obtained from Mouse 3 on ensuing days post-infection each produced a different Vlp during infection at 3 day intervals. Notably, variants that emerged from these mice may have been present in the non-clonal parent as subdominant populations according to PacBio sequencing reads (Table 1; Table S1).

### Experiment 2: antigenic variation after inoculation of serotype derived from an *in vivo* isolation

**Fig 4:**
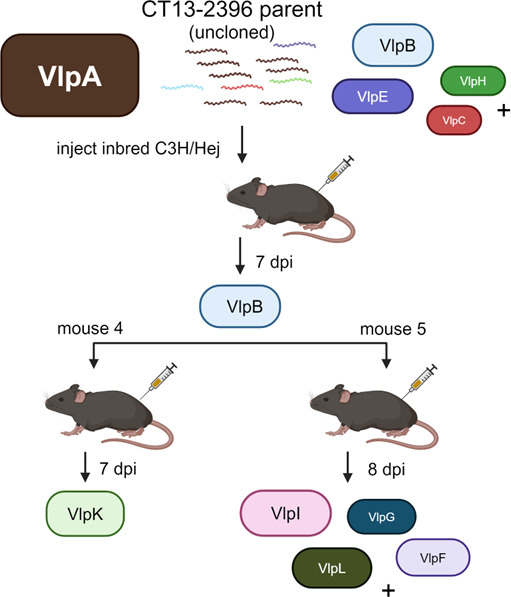
Vlp serotype determinations from Experiment 2 mouse reisolates. Strain CT13-2396 parent strain inoculated into C3H/Hej mice (*n* = 3) with one mouse yielding a reisolate identified as VlpB, which was inoculated into a second cohort of C3H/Hej mice (*n* = 3) of which two, Mice 4 and 5, produced reisolates at 7 and 8 dpi, respectively. Uncloned parent strain is illustrated as VlpA serotype majority with subdominant serotypes (B, E, H, and J) listed according to reads determined by PacBio sequencing in Table 4. Vlp genes identified as being in the expression locus are indicated by letters and are color coded with corresponding GenBank locus reference tag designations listed in Table 3. (+) indicates additional subdominant serotypes that may be present in the populations. Sequencing reads for Mouse 5 (8 dpi) reisolate with the legend for gene locus tag designations corresponding to the letters are listed in Table S1.

For this experiment, a VlpB majority serotype (determined by expression locus PCR and Sanger sequencing) that was reisolated from a mouse injected with the non-clonal parent strain at 7 dpi, was inoculated into C3H/Hej mice (*n* = 3) (Fig. 4). Two of these mice (Mouse 4 and Mouse 5) yielded blood reisolates at 7 and 8 dpi, respectively. Expression locus PCR using the prExpF/Vlp25R primer set revealed that the VlpB serotype was replaced in the Mouse 4 reisolate by a single variant, VlpK, as determined by clean Sanger sequencing (Fig. 4). Mouse 5 reisolate was a mixed population of serotypes determined by PacBio sequencing, with VlpI and VlpL represented by the highest read numbers followed by VlpF and VlpG (Fig. 4; Table S1). The sequencing data also revealed a low number of VlpB reads indicating the inoculated serotype was abated during infection (Table S1). No PCR products were generated using the prExpF/Vlp4R or prExpF/Vsp1 primer sets.

This experiment demonstrated that inoculation of a serotype derived from an *in vivo* infection resulted in the expansion of new serotypes.

### Experiment 3: reisolate derived from outbred mouse inoculation undergoes serotype variation during infection

**Fig 5:**
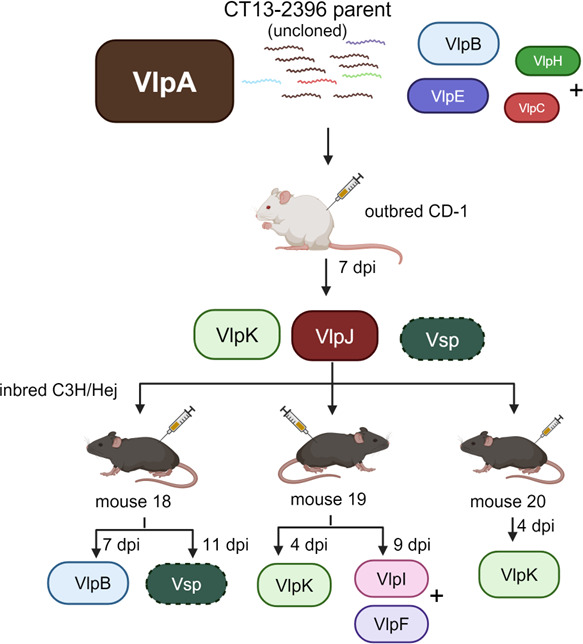
Vlp serotype determinations from Experiment 3 mouse reisolates. Strain CT13-2396 parent strain inoculated into outbred CD-1 (*n* = 3) with one mouse yielding a reisolate identifed as a mixed serotype population of VlpK, VlpJ, and Vsp. This reisolate was inoculated into a cohort of C3H/Hej (*n* = 3) mice each of which yielded reisolates as shown. Uncloned parent strain is illustrated as VlpA serotype majority with subdominant serotypes (B, E, H, and J) listed according to reads determined by PacBio sequencing in Table 4. Vlp genes identified as being in the expression locus are indicated by letters and are color coded with corresponding GenBank locus reference tag designations listed in Table 3. (+) indicates additional subdominant serotypes that may be present in the populations. Sequencing reads for the Mouse 19 (9 dpi) reisolate with the legend for gene locus tag designations corresponding to the letters are listed in Table S1.

This experiment followed the protocol of Experiment 2 except that CD-1 outbred mice (*n* = 3) were used to assess *B. miyamotoi* variation in comparison to the C3H/Hej immunocompetent inbred mouse strain. A reisolate was obtained from one CD-1 mouse at 7 dpi, and in this case, expression locus PCR products were generated using three separate reverse primers,Vsp1R, Vlp4R, and Vlp25R that were paired with prExpF (Fig. 5). Clean Sanger sequencing of each amplicon revealed that this reisolate consisted of three variants identified as Vlp delta subfamily (VlpK), Vlp alpha subfamily (VlpJ), and Vsp family (Fig. 5). The Vsp gene, AXH25_RS05675, was located on lp41, and a duplicate, AXH25_RS05390, was located on lp26. Again, no amplicon was produced using the VlpAR reverse primer, indicating the VlpA serotype was eliminated or diminished below detection.

**Fig 6:**
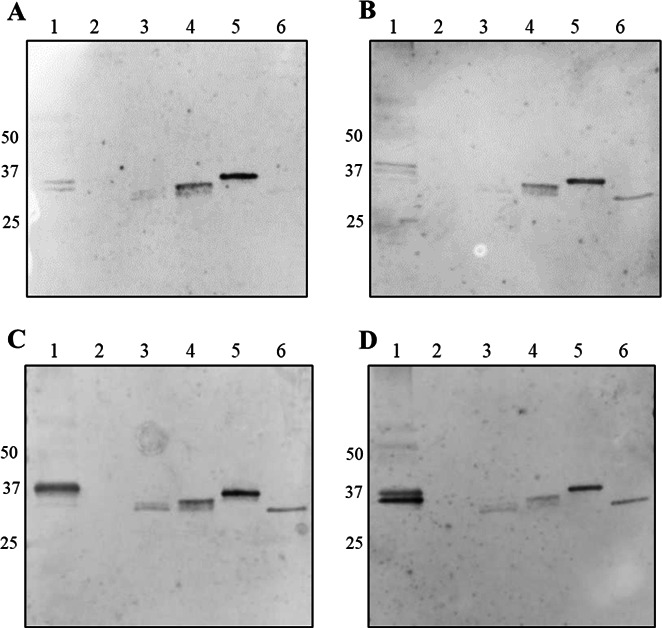
IgM immunoblots of serum from Mice 18 and 19 from Experiment 3 and Mice 13 and 14 from Experiment 4 against *B. miyamotoi* whole-cell lysate and recombinant (r) Vmps. (A) Mouse 18, (**B**) Mouse 19, (**C**) Mouse 13, and (**D**) Mouse 14. Lane 1, *B. miyamotoi* whole-cell lysate; Lane 2, rVsp1; Lane 3, rVlpD; Lane 4, rVlpB; Lane 5, rVlpC; Lane 6, rVlpA. M, molecular mass marker with kilodalton size shown between panels A and B, and left and right of panels C and D, respectively.

The mixed population of three serotype variants isolated from the CD-1 mouse was inoculated into C3H/Hej mice (*n* = 3), and spirochetes were reisolated from each mouse (Fig. 5). An isolate from Mouse 18 at 7 dpi was identified as VlpB serotype, which was not detected in the inoculum. A second isolate recovered from Mouse 18 at 11 dpi 4 days later was identified as Vsp serotype that was present in the inoculum. This result indicated that a VlpB serotype arose during Mouse 18 infection but suggested the Vsp serotype either evaded the initial antibody response and emerged later or was eliminated early and arose later possibly by a new *vsp* gene conversion recombination. IgM immunoblot for Mouse 18 serum drawn at 15 dpi showed no reactivity against recombinant Vsp1, suggesting a lack of antibody pressure against the Vsp serotype (Fig. 6A, Lane 2). IgM reactivity against recombinant VlpB was observed, suggesting that this serotype was eliminated by the antibody response during infection (Fig. 6A, Lane 4) with the emergence of Vsp serotype by 11 dpi. IgM reactivity was also observed against recombinant VlpD and VlpC, as well as bands within the correct molecular size range for Vlps in the whole-cell lysate (Fig. 6A; Lanes 1, 3, and 5). Vlps contain regions of homogeneity; therefore, antibody cross-reactivity among these proteins is commonly detected. IgM reactivity against VlpA was not observed presumably because this serotype, originally a majority serotype in the uncloned parent strain, had been eliminated during the CD-1 mouse infection and therefore was not present in the inoculum (Fig. 6A, Lane 6).

The Mouse 19 reisolate at 4 dpi was serotype VlpK (Fig. 5). VlpK was one of the serotypes inoculated, suggesting that an immune response had not yet developed to cause antigenic switching. However, by 9 dpi, a second Mouse 19 isolate was obtained and found to consist of multiple variants with the majority being serotypes VlpI and VlpF (Fig. 5; Table S1). VlpK in this reisolate was present according to the PacBio sequencing, but the reads were diminished, suggesting that the IgM response was working to eliminate this serotype. Indeed, by 15 dpi, Mouse 19 serum IgM was cross-reactive against recombinant Vlps (Fig. 6B; Lanes 1 and 4–6). An isolate obtained from Mouse 20 at 4 dpi was like that from Mouse 19 at 4 dpi, i.e., an early VlpK serotype (Fig. 5). We were unable to isolate additional *B. miyamotoi* from Mouse 20 later in the infection.

This experiment showed that serotype variation occurred in outbred mice, although observed with a limited sample size (*n* = 1), and that an alpha and delta Vlp subfamily and Vsp subfamily were isolated. Also observed was a repeat of Experiments 1 and 2 insofar as new serotypes were reisolated from infected inbred mice. The mice in this experiment produced early IgM antibody responses against recombinant Vmps, suggesting that antigenic switching occurred as a result.

### Experiment 4: clonal serotype undergoes antigenic variation during mouse infection

**Fig 7:**
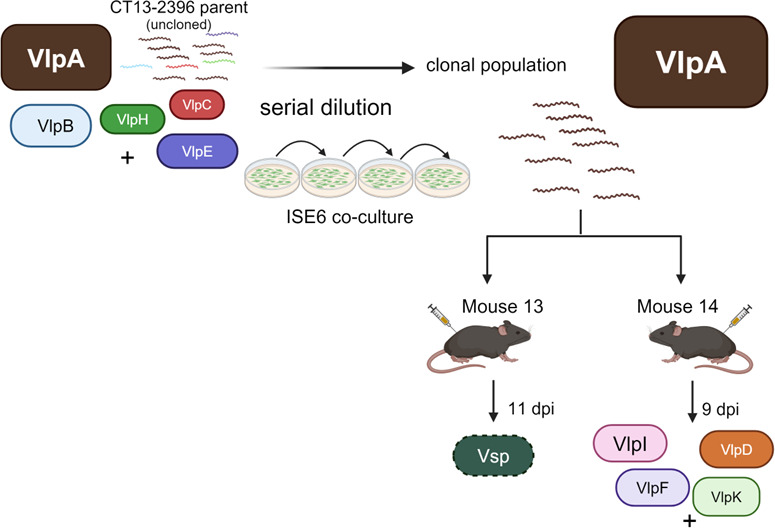
Vlp serotype determinations from Experiment 4 mouse reisolates. Uncloned parent strain is illustrated as VlpA serotype majority with subdominant serotypes (B, E, H, and J) listed according to reads determined by PacBio sequencing in Table 2. The parent strain was serially diluted in co-culture with ISE6 cells to produce VlpA serotype clonal population that was inoculated into C3H/Hej mice, yielding reisolates as shown. Uncloned parent strain is illustrated as VlpA serotype majority with subdominant serotypes (B, E, H, and J) listed according to reads determined by PacBio sequencing in Table 3. Vlp genes identified as being in the expression locus are indicated by letters and are color coded with corresponding GenBank locus reference tag designations listed in Table 3. (+) indicates additional subdominant serotypes that may be present in the populations. Sequencing reads for the Mouse 14 (9 dpi) reisolate with the legend for gene locus tag designations corresponding to the letters are listed in Table S1.

A *B. miyamotoi* clonal population was generated by serial dilution of the non-clonal parent strain used in Experiments 1–3. Population expansion in the 10^0^ spirochete wells was observed, and borreliae were collected (Fig. 7). Clonal assessement was performed by expression locus PCR using the VlpAR primer, and Sanger sequencing of the PCR product revealed a serotype population of VlpA in each of the replicates with no evidence of overlapping peaks in the profile. PCR products were not detected using the Vlp25R, Vlp4R, or Vsp1R primers. Therefore, we concluded that serial dilution produced a VlpA serotype clonal population. This result was confirmed in each of the quadruplicate serial dilution replicates.

C3H/Hej mice were inoculated with the clonal VlpA serotype with two mice producing reisolates. Like the previous experiments, expression locus PCR did not detect VlpA in the reisolates. The reisolate from Mouse 13 at 11 dpi was a Vsp serotype (Fig. 7). The reisolate from Mouse 14 at 9 dpi was a mixed population with VlpD, VlpI, VlpK, and VlpF represented by the most PacBio reads (Fig. 7; Table S1). IgM immunoblots with Mouse 13 and Mouse 14 serum at 15 dpi showed reactivity against recombinant VlpA (Fig. 6C, Lane 6; Fig. 6D, Lane 6), suggesting that anti-VlpA antibodies resulted in VlpA serotype elimination and subsequent serotype switching. Also, cross-reactive antibodies were seen against cell lysate Vlps and the other recombinant Vlps, but not for Vsp, in these immunoblots (Fig. 6C and D, Lanes 1–5).

The results of this experiment provide evidence that the clonal population injected into mice antigenically switched to a new serotype during infection by gene conversion of an archived pseudogene into the expression locus.

## DISCUSSION

The aim of this study was to determine if *B. miyamotoi* antigenically shifts *in vivo*, which is the fundamental mechanism behind RFB immune evasion seen in mammalian infections and relapsing fever patients. Clinical case descriptions of *B. miyamotoi* infections have indicated that relapsing fever occurs in relatively few cases of HTRF compared to infections by other RFB (13, 14). Our goal was to assess whether *B. miyamotoi* antigenic variation occurs during mammalian infection to gain insight into the clinical manifestations of this disease in human patients.

Strain CT13-2396 was used in this study because previous work in our laboratory found that mouse infection with strain LB-2001 was inconsistent and therefore not reliable (37).

We confirmed that the CT13-2396 parent strain used in this study incorporated VlpA (i.e., Vlp5) in the expression locus as annotated in the GenBank genomic sequence by using a reverse primer (VlpAR) to specifically amplify the VlpA gene. Although not quantifiable, the evidence pointed to VlpA being the predominant serotype in the parent strain based on (i) a distinct, strong, single PCR product upon amplification; (ii) elimination of *vlpA* from the expression site for each mouse blood reisolate as determined by PCR; (iii) IgM anti-VlpA antibodies elicited in mice early after infection; (iv) PacBio sequencing from the expression locus of every reisolate indicating absence of *vlpA* (except for a lone read in the Mouse 3 isolate at 6 dpi); and (v) serial dilution cloning consistently giving only *B. miyamotoi* VlpA clones.

However, we observed a minor amplicon band derived from the expression locus of the parent strain when the reverse primer Vlp25R was used, suggesting subdominant serotype populations were present. This observation was confirmed by PacBio sequencing of the Vlp25R-generated amplicon band from the parent strain. This result was an important factor in the interpretation of the mechanism that determined serotype variation from infected mice in the subsequent experiments and is discussed below.

We were successful using co-cultivation with ISE6 tick cells to propagate *B. miyamotoi* from small amounts of mouse blood obtained by tail nicking, presumably when borrelial density was low, whereby direct inoculation in liquid media failed to produce growth. Previous work with mouse models in our laboratory demonstrated that reisolates could be grown from blood by this method when organisms could not be detected by microscopy or PCR (37). *B. miyamotoi* blood reisolates were obtained on different days post-inoculation, depending on the individual mouse. Some mice yielded one reisolate, while some mice yielded more than one during the course of infection. We obtained reisolates from as early as 4 dpi out to 11–12 dpi. While we were successful in reisolating *B. miyamotoi* from infected mouse blood by this method, more experimentation is needed to determine patterns of relapses during mouse infection. However, the successful isolation of two or more variants from some mice suggested that *B. miyamotoi* serotype switching occurred on a recurring basis during infection.

Another observation regarding reisolates was that expression locus PCR indicated some appeared clonal, i.e., by “clean” Sanger sequencing, and some were mixed variant populations. Reisolates that resulted in expression locus amplicons that indicated a clear majority serotype by clean Sanger sequencing were not subjected to PacBio sequencing; therefore, the possibility for minority subpopulations to be present cannot be discounted. Whether the non-clonal reisolations we found resulted from spontaneous switching during culture expansion or from multiple recombinations during infection remains to be explicated. However, it was clear from all the experimental reisolates obtained that the VlpA majority population was not detectable by expression locus PCR, demonstrating that the predominant parent serotype was likely eliminated or severely reduced.

Experiments 1, 2, 3, and 4 served as biological repeats in that different variants from the parent were reisolated, but each experiment had a slightly different wrinkle in the approach. Experiment 1 showed that new majority population serotypes differing from the parent strain could be reisolated from blood of multiple mice. Experiment 2 mice were inoculated with a blood reisolate with a majority serotype different from the parent that resulted in new variants. Experiment 3 also employed a blood reisolate from an outbred mouse that was a mixed serotype population of three Vmps for mouse inoculations. Experiment 4 was distinct in that a clonal population was administered to mice and therefore was best suited to determine whether new variants arose by gene conversion or resulted from survival of subdominant populations. The data from Experiment 4 strongly indicate antigenic switching by gene conversion occurred rather than expansion of minority subpopulations.

Each experiment yielded some blood isolates that were of mixed serotype populations as determined by PacBio amplicon sequencing. The number of reads corresponding to individual archived pseudogenes provided an indication of both the serotypes represented and dominance in the population. All variants that were reisolated during the experimental infections appeared to be present in the non-clonal parent as subdominant populations according to PacBio sequencing, although VlpL, VlpF, VlpG, and VlpJ among others were listed at very low frequencies, i.e., from 1 to 41 reads (Table 3).

Our previous work proposed two hypotheses for antigenic variation (35). The new variant hypothesis proposed that anti-Vmp antibodies eliminate the corresponding serotypes expressing those Vmps, resulting in antigenic switching by gene conversion to generate a new Vmp serotype population. The subdominant variant model proposed that anti-Vmp antibodies eliminate corresponding serotypes expressing those Vmps but have no effect on a low-frequency variant population that is present which subsequently expands. The two mouse blood reisolates resulting from Experiment 4 clonal inoculation found a Vsp serotype in one mouse and a mixed Vlp serotype population in the other, providing evidence for the new variant hypothesis.

There appeared to be a bias for certain archived genes, mainly subfamily delta, to be translocated to the expression locus as the variants from all the experiments indicated a preference for some archived pseudogenes over others; i.e., only Vlps B–L were represented, while other pseudogenes were absent or possibly of low frequency (Fig. 1; Table S1). Indeed, Vlp25R primer was not optimal for amplification of strain CT13-2396 subfamily gamma genes, and therefore, they may have been unseen. Therefore, it remains to be determined whether certain pseudogenes are preferred for expression, perhaps by a translocation mechanism driven by flanking sequences as reported for other RFBs (29, 30, 38, 39).

Some archived pseudogenes that were expressed are present in duplicate copies on plasmids lp41 and lp26 (Table 1). Therefore, it could not be ascertained from which plasmid the translocated gene in the expression locus originated, although it would be likely that the lp41 genes may be preferred for switching based on being on the same plasmid as the expression locus.

Further studies will be required to address the limitations encountered with these findings. For example, expression locus PCR was a qualitative assay. Therefore, the level of subdominant variants was not quantifiable, and the frequency that spontaneous recombination may occur during culture growth is unknown. Mouse reisolates were not recovered on consistent days post-inoculation. Therefore, work defining relapses during the course of infection may depend on the *B. miyamotoi* strain, the host mouse strain, and improved blood reisolation methods. Additionally, because we could not test spirochetes directly from blood and were required to expand them in tick cell co-culture, there could be a bias for certain serotypes that grow better in that environment. Finally, future studies should focus on determining optimal growth reisolation conditions for particular *B. miyamotoi* strains to be used in mouse infection experiments and to improve PCR and sequencing methods for identification of serotypes directly in blood samples. For example, with the addition of multiple *B. miyamotoi* genome sequences available on public databases, designing individual PCR primers specific for each *vmp* pseudogene would allow for a more comprehensive and systematic approach to determine antigenic variation in an infection model.

In conclusion, the data showed that *B. miyamotoi* reisolated from infected mice antigenically switched to a new predominant serotype from the inoculated serotype. Further studies regarding the mechanisms driving the generation of new variants for *B. miyamotoi in vivo* will aid in understanding the clinical presentations of HTRF in humans for this emerging tick-borne pathogen.

## MATERIALS AND METHODS

### *Borrelia miyamotoi* culture conditions and reisolation from mouse blood by co-cultivation with ISE6 tick cells

*Borrelia miyamotoi* strain CT13-2396 was used for all mouse inoculation experiments. *B. miyamotoi* strain CT13–2396 was isolated from an infected *Ixodes scapularis* nymph collected in Connecticut, USA (31). Cultures were initiated from frozen low-passage-number (<10 passages) glycerol stocks and cultivated in modified Barbour-Stoenner-Kelly medium (BSK-R) (40) at 34°C in capped tubes until they reached the late log phase, approximately from 1 × 10^6^ to 1 × 10^7^ cells/mL. Prior to the mouse inoculations, cultures were enumerated using Cellometer disposable counting chambers (Nexcelom, Lawrence, MA, USA).

ISE6 tick cells were grown to confluency on Corning Costar flat-bottom tissue culture plates (Thermo Fisher Scientific, Waltham, MA, USA) in L15B media, sealed with PCR plate sealing adhesive under the plate lid and wrapped in parafilm, with incubation at 34°C (37, 41). When co-culturing with *B. miyamotoi*, L15B media was diluted with sterile water to make L15B-300 media as described by Munderloh et al. (42).

*B. miyamotoi* was reisolated from mouse blood by co-cultivation with ISE6 cells, as described previously (37). Tail nick mouse blood (5 µL) was suspended in 45 µL phosphate-buffered saline (PBS) with 10 µL of blood suspension inoculated onto ISE6 cells in 12-well plates containing 2 mL L15B-300 and 20% BSK-R. After 7 days, cultures were checked for spirochete growth by darkfield microscopy, and media were refreshed as follows. Media were removed from each well, spun at 8,000 × *g* for 5 min to pellet *Borrelia*. During the spin, fresh L15B-300 was added to the ISE6 cells to retain hydration and viability. Pelleted bacteria were resuspended in fresh BSK-R, so the final volume per well would be 20% BSK-R, and were returned to the original ISE6 wells. This process was repeated every 7–10 days as required until *Borrelia* spp. were at an appropriate density for freezing glycerol stock cultures or collecting bacterial pellets (≥5 × 10^6^/mL). Bacterial pellets were collected by removing approximately 1 mL of media from the well and centrifuging at 8,000 × *g* for 5 min. The supernatant was decanted; the pellet was washed in sterile PBS; and the centrifugation was repeated. The supernatant was removed and the pellet was stored at −80°C.

A *B. miyamotoi* clonal isolate was obtained by serially diluting a parent CT13-2396 isolate on confluent ISE6 cells in a 96-well plate down to 10^−1^/well with incubation at 34°C, 5% CO_2_, for 10–17 days until population expansion was observed in the 10^0^ wells. When the population in a well reached a density of ~1 × 10^6^ spirochetes/mL, the contents were transferred to a 12-well plate with ISE6 cells and incubated for continued growth, and *B. miyamotoi* spp. were collected as described above. Serial dilution was performed in quadruplicate.

### *B. miyamotoi* inoculations in mice

Mice used in these studies were 10- to 18-week-old female C3H/HeJ mice or CD-1 mice obtained from Charles Rivers Laboratories (Wilmington, MA, USA). Mice were infected by subcutaneous needle inoculation with 1 × 10^6^ spirochetes in their respective growth medium. After needle inoculation, mice underwent tail nicks either daily or on a staggered schedule to comply with Institutional Animal Care and Use Committee protocols (Table 4). Mice were anesthetized with isofluorane, and the tip of their tail was cleaned with 70% ethanol and removed with sterile scissors. Blood was expressed through gentle pressure on the tail with 5 µL collected and resuspended in 45 µL of sterile PBS. Pressure was applied to stop the bleeding, and the mice were returned to their cage. If the mice were on a staggered bleeding schedule, one mouse in each group was bled each day, alternating mice so an individual mouse was bled every 3 days. At the end of each experiment, mice were terminally bled by cardiac puncture, and the sera were separated from the blood using either a serum separator tube (BD, Franklin Lakes, NJ, USA) spun at 6,000 × *g* for 10 min or a microcentrifuge tube spun at 8,000 × *g* for 15 min. Table 1 summarizes the *B. miyamotoi* mouse infections for each experiment detailed in the Results.

### Expression locus PCR and DNA sequencing

The parent strain and each isolate were subjected to expression locus PCR using the forward primer prExpF, specific for the unique expression promoter, paired with each of the following reverse primers: VlpAR, Vlp25R, Vlp4R, and Vsp1R (Table 2). The latter three primers were designed from strain LB-2001 and used in a previous study (35). The Vlp25R reverse internal primer was designed from a conserved region of 25 LB-2001 *vlp* genes after nucleotide sequence alignment using MegAlign Muscle algorithm (Lasergene; DNASTAR, Madison, WI, USA) approximately 1 kb into the coding sequence. The 25-strain LB-2001 Vlps were from subfamilies beta, delta, and gamma. Vlp25R alignment with strain CT13-2396 shows that 21 *vlp* genes have no mismatches or no more than two mismatches with the delta subfamily (Fig. S1).

The remaining four Vlps of subfamily alpha in strain LB-2001 were aligned as above to design an internal reverse primer, Vlp4R (35). Vlp4R aligned with the five alpha subfamily *vlps* in strain CT13-2396 with no mismatches (Fig. S2). The VlpAR reverse internal primer was designed from the CT13-2396 strain GenBank sequence (AXH25_04655, annotated as variable large protein 5) and was used and termed VlpC2R in a previous study (35).

DNA templates were prepared from 1 mL *B. miyamotoi* culture pellets (approximately 1 × 10^7^

cells) resuspended in sterile nanopure water (50 µL) and boiled for 5 min with 0.5–1.0 µL used in

the PCR. PCRs were performed in 20 µL volume with 0.1 µM primer and 2× CloneAmp HiFi PCR Premix (TaKaRa, San Jose, CA, USA). Reaction parameters were 95°C for 2 min (one cycle); 95°C for 10 s, 55°C for 20 s, 72°C for 30 s (35 cycles); and 72°C for 5 min (one cycle). PCRs using the Vlp25R primer used a 50°C annealing temperature. Amplicons were run on 1% Tris-acetate-EDTA agarose gels and were purified using QIAquick gel extraction kit (Qiagen, Hilden, Germany) prior to DNA sequencing.

Amplicon PacBio libraries were prepared using the SMRTbell Express Template Prep Kit version 3.0 (Pacific Biosciences, Menlo Park, CA, USA) and barcoded using the Barcoded Overhang Adapter Kit 8A (Pacific Biosciences). Library quality was confirmed via Qubit (Thermo Fisher Scientific) and Femto Pulse (Agilent, Santa Clara, CA, USA), and an equimolar pool was made. The pooled libraries were prepared for sequencing using the Sequel Binding kit version 3.1 (Pacific Biosciences) and sequenced on the Sequel II instrument. Sequencing read data were analyzed using CLC Genomics Workbench v.23.0.4 software (Qiagen).

Sanger DNA amplicon sequencing was performed using Big Dye Terminator v.3.1 Sequencing Standard Kit (Thermo Fisher Scientific), and reactions were run on Applied Biosystems 3500XL Genetic Analyzer (Thermo Fisher Scientific). Sequencing data were analyzed using Lasergene 17 SeqMan Pro software (DNASTAR).

### Recombinant variable major proteins and Western blotting

VlpB, VlpC, and VlpD genes were amplified by PCR from strain CT13-2396 genomic DNA using the primers listed in Table 2 and were cloned into the expression plasmid pETite N-His vector (Lucigen, Middleton, WI, USA). Plasmids were transformed into *E. coli* BL21 (DE3), and recombinant proteins were expressed and purified from BL21 bacterial culture using a QiaExpress Ni-NTA FastStart kit (Qiagen, Valencia, CA, USA) as described previously (43). Recombinant Vsp1 (without the signal peptide) was produced in the same manner with pETite C-His vector with primers listed in Table 2. Recombinant VlpA (VlpC2) was produced as described previously (44). Protein concentration determinations were performed by bicinchoninic acid assay following the manufacturer’s instructions (Thermo Fisher Scientific).

*B. miyamotoi* whole-cell lysates were prepared by pelleting 1 × 10^8^ *B. miyamotoi* cells and decanting the spent media, followed by 2× PBS washes. Washed cell pellets were resuspended in 100 µL of PBS and 100 µL of 2× SDS-PAGE sample loading buffer for a final concentration of 5 × 10^5^ cells/µL. Twenty microliters of whole-cell lysate was loaded per lane.

Whole-cell *B. miyamotoi* protein lysate or recombinant proteins (100 ng/well) were separated by SDS-PAGE on AnykD mini-protean gels (Bio-Rad, Hercules, CA, USA). Proteins were transferred to polyvinylidene difluoride membranes using the iBlot transfer stack (Invitrogen, Waltham, MA, USA) and blocked overnight in SuperBlock TBS blocking buffer (Thermo Fisher Scientific). Membranes were washed three times for 5 min each with 1× Tris-buffered saline with 0.05% Tween 20 (TBS-T) followed by room temperature incubation with mouse sera at a 1:200 dilution in TBS-T for 1 hour. Membrane washes were repeated and probed with goat anti-mouse IgM peroxidase-conjugated antibody diluted at 1:30,000 for 40 min. Membranes were washed and developed with Amersham ECL Western blotting detection reagent (Cytiva, Marlborough, MA, USA) and imaged on an Azure Imager (Azure Biosystems, Dublin, CA, USA). Control blots were performed using secondary antibody, only finding that non-specific binding did not occur.

## SUPPLEMENTAL MATERIAL

Supplemental material iai.00484-24-s0001.pdf Table S1; Fig. S1 and S2.

ASM does not own the copyrights to Supplemental Material that may be linked to, or accessed through, an article. The authors have granted ASM a non-exclusive, world-wide license to publish the Supplemental Material files. Please contact the corresponding author directly for reuse.

## References

[1] Fukunaga M, Koreki Y. 1995. The flagellin gene of Borrelia miyamotoi sp. nov. and its phylogenetic relationship among Borrelia species. FEMS Microbiol Lett 134:255–258. doi:10.1111/j.1574-6968.1995.tb07947.x 8586277

[2] Fukunaga M, Takahashi Y, Tsuruta Y, Matsushita O, Ralph D, McClelland M, Nakao M. 1995. Genetic and phenotypic analysis of Borrelia miyamotoi sp. nov., isolated from the ixodid tick Ixodes persulcatus, the vector for Lyme disease in Japan. Int J Syst Bacteriol 45:804–810. doi:10.1099/00207713-45-4-804 7547303

[3] Platonov AE, Karan LS, Kolyasnikova NM, Makhneva NA, Toporkova MG, Maleev VV, Fish D, Krause PJ. 2011. Humans infected with relapsing fever spirochete Borrelia miyamotoi, Russia. Emerg Infect Dis 17:1816–1823. doi:10.3201/eid1710.101474 22000350 PMC3310649

[4] Hovius JWR, de Wever B, Sohne M, Brouwer MC, Coumou J, Wagemakers A, Oei A, Knol H, Narasimhan S, Hodiamont CJ, Jahfari S, Pals ST, Horlings HM, Fikrig E, Sprong H, van Oers MHJ. 2013. A case of meningoencephalitis by the relapsing fever spirochaete Borrelia miyamotoi in Europe. Lancet 382:658. doi:10.1016/S0140-6736(13)61644-X 23953389 PMC3987849

[5] Chowdri HR, Gugliotta JL, Berardi VP, Goethert HK, Molloy PJ, Sterling SL, Telford SR. 2013. Borrelia miyamotoi infection presenting as human granulocytic anaplasmosis: a case report. Ann Intern Med 159:21–27. doi:10.7326/0003-4819-159-1-201307020-00005 23817701

[6] Gugliotta JL, Goethert HK, Berardi VP, Telford SR. 2013. Meningoencephalitis from Borrelia miyamotoi in an immunocompromised patient. N Engl J Med 368:240–245. doi:10.1056/NEJMoa1209039 23323900 PMC4018741

[7] Sato K, Takano A, Konnai S, Nakao M, Ito T, Koyama K, Kaneko M, Ohnishi M, Kawabata H. 2014. Human infections with Borrelia miyamotoi, Japan. Emerg Infect Dis 20:1391–1393. doi:10.3201/eid2008.131761 25061761 PMC4111186

[8] Molloy PJ, Telford SR 3rd, Chowdri HR, Lepore TJ, Gugliotta JL, Weeks KE, Hewins ME, Goethert HK, Berardi VP. 2015. Borrelia miyamotoi disease in the Northeastern United States: a case series. Ann Intern Med 163:91–98. doi:10.7326/M15-0333 26053877

[9] Mead PS. 2023. Nomenclature for human infections caused by relapsing fever borrelia. Emerg Infect Dis 29:1084. doi:10.3201/eid2905.230195 37081502 PMC10124633

[10] Telford SR 3rd, Goethert HK, Molloy PJ, Berardi VP, Chowdri HR, Gugliotta JL, Lepore TJ. 2015. Borrelia miyamotoi disease: neither Lyme disease nor relapsing fever. Clin Lab Med 35:867–882. doi:10.1016/j.cll.2015.08.002 26593262 PMC4662080

[11] Krause PJ, Barbour AG. 2015. Borrelia miyamotoi: the newest infection brought to us by deer ticks. Ann Intern Med 163:141–142. doi:10.7326/M15-1219 26053967

[12] Koetsveld J, Kolyasnikova NM, Wagemakers A, Toporkova MG, Sarksyan DS, Oei A, Platonov AE, Hovius JW. 2017. Development and optimization of an in vitro cultivation protocol allows for isolation of Borrelia miyamotoi from patients with hard tick-borne relapsing fever. Clin Microbiol Infect 23:480–484. doi:10.1016/j.cmi.2017.01.009 28110053

[13] McCormick DW, Brown CM, Bjork J, Cervantes K, Esponda-Morrison B, Garrett J, Kwit N, Mathewson A, McGinnis C, Notarangelo M, Osborn R, Schiffman E, Sohail H, Schwartz AM, Hinckley AF, Kugeler KJ. 2023. Characteristics of hard tick relapsing fever caused by Borrelia miyamotoi, United States, 2013-2019. Emerg Infect Dis 29:1719–1729. doi:10.3201/eid2909.221912 37610298 PMC10461660

[14] Hoornstra D, Azagi T, van Eck JA, Wagemakers A, Koetsveld J, Spijker R, Platonov AE, Sprong H, Hovius JW. 2022. Prevalence and clinical manifestation of Borrelia miyamotoi in Ixodes ticks and humans in the northern hemisphere: a systematic review and meta-analysis. Lancet Microbe 3:e772–e786. doi:10.1016/S2666-5247(22)00157-4 36113496

[15] Burde J, Bloch EM, Kelly JR, Krause PJ. 2023. Human Borrelia miyamotoi Infection in North America. Pathogens 12:553. doi:10.3390/pathogens12040553 37111439 PMC10145171

[16] Lopez J, Hovius JW, Bergström S. 2021. Pathogenesis of relapsing fever. Curr Issues Mol Biol 42:519–550. doi:10.21775/cimb.042.519 33372163 PMC8756760

[17] Barbour AG, Hayes SF. 1986. Biology of Borrelia species. Microbiol Rev 50:381–400. doi:10.1128/mr.50.4.381-400.1986 3540570 PMC373079

[18] Schwan TG. 2021. Vector specificity of the relapsing fever spirochete Borrelia hermsii (Spirochaetales: Borreliaceae) for the tick Ornithodoros hermsi (Acari: Argasidae) involves persistent infection of the salivary glands. J Med Entomol 58:1926–1930. doi:10.1093/jme/tjab060 33855354 PMC8285016

[19] Stoenner HG, Dodd T, Larsen C. 1982. Antigenic variation of Borrelia hermsii. J Exp Med 156:1297–1311. doi:10.1084/jem.156.5.1297 7130900 PMC2186838

[20] Coffey EM, Eveland WC. 1967. Experimental relapsing fever initiated by Borrelia hermsi. II. Sequential appearance of major serotypes in the rat. J Infect Dis 117:29–34. doi:10.1093/infdis/117.1.29 5338694

[21] Hinnebusch BJ, Barbour AG, Restrepo BI, Schwan TG. 1998. Population structure of the relapsing fever spirochete Borrelia hermsii as indicated by polymorphism of two multigene families that encode immunogenic outer surface lipoproteins. Infect Immun 66:432–440. doi:10.1128/IAI.66.2.432-440.1998 9453591 PMC107923

[22] Carter CJ, Bergström S, Norris SJ, Barbour AG. 1994. A family of surface-exposed proteins of 20 kilodaltons in the genus Borrelia. Infect Immun 62:2792–2799. doi:10.1128/iai.62.7.2792-2799.1994 8005669 PMC302883

[23] Restrepo BI, Carter CJ, Barbour AG. 1994. Activation of a vmp pseudogene in Borrelia hermsii: an alternate mechanism of antigenic variation during relapsing fever. Mol Microbiol 13:287–299. doi:10.1111/j.1365-2958.1994.tb00423.x 7984108

[24] Pennington PM, Cadavid D, Barbour AG. 1999. Characterization of VspB of Borrelia turicatae, a major outer membrane protein expressed in blood and tissues of mice. Infect Immun 67:4637–4645. doi:10.1128/IAI.67.9.4637-4645.1999 10456910 PMC96788

[25] Plasterk RH, Simon MI, Barbour AG. 1985. Transposition of structural genes to an expression sequence on a linear plasmid causes antigenic variation in the bacterium Borrelia hermsii. Nature New Biol 318:257–263. doi:10.1038/318257a0 4069202

[26] Barbour AG, Carter CJ, Sohaskey CD. 2000. Surface protein variation by expression site switching in the relapsing fever agent Borrelia hermsii. Infect Immun 68:7114–7121. doi:10.1128/IAI.68.12.7114-7121.2000 11083837 PMC97822

[27] Barbour AG, Burman N, Carter CJ, Kitten T, Bergström S. 1991. Variable antigen genes of the relapsing fever agent Borrelia hermsii are activated by promoter addition. Mol Microbiol 5:489–493. doi:10.1111/j.1365-2958.1991.tb02132.x 2041480

[28] Barbour AG, Carter CJ, Burman N, Freitag CS, Garon CF, Bergström S. 1991. Tandem insertion sequence-like elements define the expression site for variable antigen genes of Borrelia hermsii. Infect Immun 59:390–397. doi:10.1128/iai.59.1.390-397.1991 1987053 PMC257753

[29] Dai Q, Restrepo BI, Porcella SF, Raffel SJ, Schwan TG, Barbour AG. 2006. Antigenic variation by Borrelia hermsii occurs through recombination between extragenic repetitive elements on linear plasmids. Mol Microbiol 60:1329–1343. doi:10.1111/j.1365-2958.2006.05177.x 16796672 PMC5614446

[30] Kitten T, Barrera AV, Barbour AG. 1993. Intragenic recombination and a chimeric outer membrane protein in the relapsing fever agent Borrelia hermsii. J Bacteriol 175:2516–2522. doi:10.1128/jb.175.9.2516-2522.1993 7683020 PMC204552

[31] Kingry LC, Replogle A, Batra D, Rowe LA, Sexton C, Dolan M, Connally N, Petersen JM, Schriefer ME. 2017. Toward a complete north american Borrelia miyamotoi genome. Genome Announc 5:e01557-16. doi:10.1128/genomeA.01557-16 28153903 PMC5289689

[32] Barbour AG. 2016. Multiple and diverse vsp and vlp sequences in Borrelia miyamotoi, a hard tick-borne zoonotic pathogen. PLoS One 11:e0146283. doi:10.1371/journal.pone.0146283 26785134 PMC4718594

[33] Hoornstra D, Kuleshov KV, Fingerle V, Hepner S, Wagemakers A, Strube C, Castillo-Ramírez S, Bockenstedt LK, Telford SR, Sprong H, Platonov AE, Margos G, Hovius JW. 2024. Combining short- and long-read sequencing unveils geographically structured diversity in Borrelia miyamotoi. iScience 27:110616. doi:10.1016/j.isci.2024.110616 39262806 PMC11388275

[34] Wagemakers A, Koetsveld J, Narasimhan S, Wickel M, Deponte K, Bleijlevens B, Jahfari S, Sprong H, Karan LS, Sarksyan DS, van der Poll T, Bockenstedt LK, Bins AD, Platonov AE, Fikrig E, Hovius JW. 2016. Variable major proteins as targets for specific antibodies against Borrelia miyamotoi. J Immunol 196:4185–4195. doi:10.4049/jimmunol.1600014 27076681 PMC5008243

[35] Gilmore RD, Armstrong BA, Brandt KS, Van Gundy TJ, Hojgaard A, Lopez JE, Kneubehl AR. 2023. Analysis of variable major protein antigenic variation in the relapsing fever spirochete, Borrelia miyamotoi, in response to polyclonal antibody selection pressure. PLoS One 18:e0281942. doi:10.1371/journal.pone.0281942 36827340 PMC9955969

[36] Gilmore RD, Mikula S, Harris EK, Van Gundy TJ, Goodrich I, Brandt KS. 2021. Borrelia miyamotoi strain LB-2001 retains plasmids and infectious phenotype throughout continuous culture passages as evaluated by multiplex PCR. Ticks Tick Borne Dis 12:101587. doi:10.1016/j.ttbdis.2020.101587 33074149 PMC10898610

[37] Armstrong BA, Brandt KS, Goodrich I, Gilmore RD. 2023. Evaluation of immunocompetent mouse models for Borrelia miyamotoi infection. Microbiol Spectr 11:e0430122. doi:10.1128/spectrum.04301-22 36715531 PMC10100797

[38] Penningon PM, Cadavid D, Bunikis J, Norris SJ, Barbour AG. 1999. Extensive interplasmidic duplications change the virulence phenotype of the relapsing fever agent Borrelia turicatae. Mol Microbiol 34:1120–1132. doi:10.1046/j.1365-2958.1999.01675.x 10594835

[39] Restrepo BI, Kitten T, Carter CJ, Infante D, Barbour AG. 1992. Subtelomeric expression regions of Borrelia hermsii linear plasmids are highly polymorphic. Mol Microbiol 6:3299–3311. doi:10.1111/j.1365-2958.1992.tb02198.x 1484486

[40] Replogle AJ, Sexton C, Young J, Kingry LC, Schriefer ME, Dolan M, Johnson TL, Connally NP, Padgett KA, Petersen JM. 2021. Isolation of Borrelia miyamotoi and other Borreliae using a modified BSK medium. Sci Rep 11:1926. doi:10.1038/s41598-021-81252-1 33479281 PMC7820315

[41] Munderloh UG, Liu Y, Wang M, Chen C, Kurtti TJ. 1994. Establishment, maintenance and description of cell lines from the tick Ixodes scapularis. J Parasitol 80:533–543. 8064520

[42] Munderloh UG, Jauron SD, Fingerle V, Leitritz L, Hayes SF, Hautman JM, Nelson CM, Huberty BW, Kurtti TJ, Ahlstrand GG, Greig B, Mellencamp MA, Goodman JL. 1999. Invasion and intracellular development of the human granulocytic ehrlichiosis agent in tick cell culture. J Clin Microbiol 37:2518–2524. doi:10.1128/JCM.37.8.2518-2524.1999 10405394 PMC85271

[43] Brandt KS, Gilmore RD. 2017. Immunization of mice with Borrelia burgdorferi lp54 gene encoded recombinant proteins does not provide protection against tick transmitted infectious challenge. Vaccine (Auckl) 35:5310–5313. doi:10.1016/j.vaccine.2017.08.054 PMC7041491 28867507

[44] Harris EK, Harton MR, de Mello Marques MA, Belisle JT, Molins CR, Breuner N, Wormser GP, Gilmore RD. 2019. Immunoproteomic analysis of Borrelia miyamotoi for the identification of serodiagnostic antigens. Sci Rep 9:16808. doi:10.1038/s41598-019-53248-5 31727932 PMC6856195

