# Supplementary material for: Borrelia miyamotoi in vivo antigenic variation demonstrated by serotype reisolations from infected mice
Source: Infect Immun. 2025 Mar 7;93(4):e00484-24. doi: 10.1128/iai.00484-24 (PMC11977308; doi:10.1128/iai.00484-24)
Supplement: Supplemental material — Table S1; Fig. S1 and S2. [file iai.00484-24-s0001.pdf]

Mouse 1: 7dpi experiment 1

CT13-2396 Vlp locus		Reads
AXH25_RS05650↻	VlpB	61095
AXH25_RS05665↻	VlpD	15230
AXH25_RS05680↻	VlpF	1438
AXH25_RS05465↻	VlpI	896
AXH25_RS05490↻		289
AXH25_RS05670↻	VlpE	281
AXH25_RS05265↻		82
AXH25_RS05655↻	VlpC	62
AXH25_RS05195↻	VlpK	61
AXH25_RS05370↻	VlpH	48
AXH25_RS05510↻		42
AXH25_RS05500↻		35
AXH25_RS05495↻		8
AXH25_RS05270↻		7
AXH25_RS05285↻	VlpL	4
AXH25_RS05250↻		1
AXH25_RS05375↻		1
AXH25_RS05395↻		1
AXH25_RS05085↻		0
AXH25_RS05190↻		0
AXH25_RS05255↻		0
AXH25_RS05260↻		0
AXH25_RS05280↻		0
AXH25_RS05290↻		0
AXH25_RS05350↻		0
AXH25_RS05355↻	VlpG	0
AXH25_RS05475↻		0
AXH25_RS05480↻	VlpJ	0
AXH25_RS05485↻		0
AXH25_RS05505↻		0
AXH25_RS05645↻	**VlpA**	0
AXH25_RS05660↻		0
AXH25_RS05685↻		0

Mouse 2: 8dpi experiment 1

CT13-2396 Vlp locus		Reads
AXH25_RS05465↻	VlpI	66949
AXH25_RS05195↻	VlpK	12779
AXH25_RS05510↻		169
AXH25_RS05665↻	VlpD	89
AXH25_RS05370↻	VlpH	46
AXH25_RS05680↻	VlpF	40
AXH25_RS05655↻	VlpC	36
AXH25_RS05500↻		35
AXH25_RS05650↻	VlpB	33
AXH25_RS05670↻	VlpE	25
AXH25_RS05495↻		18
AXH25_RS05490↻		15
AXH25_RS05265↻		14
AXH25_RS05085↻		0
AXH25_RS05190↻		0
AXH25_RS05250↻		0
AXH25_RS05255↻		0
AXH25_RS05260↻		0
AXH25_RS05270↻		0
AXH25_RS05280↻		0
AXH25_RS05285↻	VlpL	0
AXH25_RS05290↻		0
AXH25_RS05350↻		0
AXH25_RS05355↻	VlpG	0
AXH25_RS05375↻		0
AXH25_RS05395↻		0
AXH25_RS05475↻		0
AXH25_RS05480↻	VlpJ	0
AXH25_RS05485↻		0
AXH25_RS05505↻		0
AXH25_RS05645↻	**VlpA**	0
AXH25_RS05660↻		0
AXH25_RS05685↻		0

Mouse 3: 6dpi experiment 1

CT13-2396 Vlp locus		Reads
AXH25_RS05665↻	VlpD	10107
AXH25_RS05650↻	VlpB	9337
AXH25_RS05465↻	VlpI	199
AXH25_RS05670↻	VlpE	34
AXH25_RS05510↻		19
AXH25_RS05195↻	VlpK	10
AXH25_RS05265↻		3
AXH25_RS05500↻		3
AXH25_RS05655↻	VlpC	3
AXH25_RS05370↻	VlpH	2
AXH25_RS05680↻	VlpF	2
AXH25_RS05285↻	VlpL	1
AXH25_RS05490↻		1
AXH25_RS05645↻	**VlpA**	1
AXH25_RS05085↻		0
AXH25_RS05190↻		0
AXH25_RS05250↻		0
AXH25_RS05255↻		0
AXH25_RS05260↻		0
AXH25_RS05270↻		0
AXH25_RS05280↻		0
AXH25_RS05290↻		0
AXH25_RS05350↻		0
AXH25_RS05355↻	VlpG	0
AXH25_RS05375↻		0
AXH25_RS05395↻		0
AXH25_RS05475↻		0
AXH25_RS05480↻	VlpJ	0
AXH25_RS05485↻		0
AXH25_RS05495↻		0
AXH25_RS05505↻		0
AXH25_RS05660↻		0
AXH25_RS05685↻		0

Mouse 5: 8dpi experiment 2

CT13-2396 Vlp locus		Reads
AXH25_RS05465↻	VlpI	39175
AXH25_RS05285↻	VlpL	20585
AXH25_RS05680↻	VlpF	4859
AXH25_RS05355↻	VlpG	3062
AXH25_RS05650↻	VlpB	152
AXH25_RS05670↻	VlpE	75
AXH25_RS05500↻		65
AXH25_RS05265↻		39
AXH25_RS05655↻	VlpC	27
AXH25_RS05370↻	VlpH	23
AXH25_RS05665↻	VlpD	22
AXH25_RS05195↻	VlpK	16
AXH25_RS05510↻		12
AXH25_RS05490↻		8
AXH25_RS05495↻		6
AXH25_RS05395↻		5
AXH25_RS05270↻		1
AXH25_RS05085↻		0
AXH25_RS05190↻		0
AXH25_RS05250↻		0
AXH25_RS05255↻		0
AXH25_RS05260↻		0
AXH25_RS05280↻		0
AXH25_RS05290↻		0
AXH25_RS05350↻		0
AXH25_RS05375↻		0
AXH25_RS05475↻		0
AXH25_RS05480↻	VlpJ	0
AXH25_RS05485↻		0
AXH25_RS05505↻		0
AXH25_RS05645↻	**VlpA**	0
AXH25_RS05660↻		0
AXH25_RS05685↻		0

Mouse 19: 9dpi experiment 3

CT13-2396 Vlp locus		Reads
AXH25_RS05465↻	VlpI	88616
AXH25_RS05680↻	VlpF	64574
AXH25_RS05285↻	VlpL	7963
AXH25_RS05195↻	VlpK	2033
AXH25_RS05670↻	VlpE	270
AXH25_RS05650↻	VlpB	178
AXH25_RS05395↻		113
AXH25_RS05490↻		107
AXH25_RS05655↻	VlpC	106
AXH25_RS05370↻	VlpH	97
AXH25_RS05510↻		74
AXH25_RS05665↻	VlpD	61
AXH25_RS05495↻		37
AXH25_RS05500↻		37
AXH25_RS05265↻		20
AXH25_RS05270↻		19
AXH25_RS05355↻	VlpG	1
AXH25_RS05475↻		1
AXH25_RS05085↻		0
AXH25_RS05190↻		0
AXH25_RS05250↻		0
AXH25_RS05255↻		0
AXH25_RS05260↻		0
AXH25_RS05280↻		0
AXH25_RS05290↻		0
AXH25_RS05350↻		0
AXH25_RS05375↻		0
AXH25_RS05480↻	VlpJ	0
AXH25_RS05485↻		0
AXH25_RS05505↻		0
AXH25_RS05645↻	**VlpA**	0
AXH25_RS05660↻		0
AXH25_RS05685↻		0

Mouse 14: 9dpi experiment 4

CT13-2396 Vlp locus		Reads
AXH25_RS05665↻	VlpD	181695
AXH25_RS05465↻	VlpI	115136
AXH25_RS05195↻	VlpK	57329
AXH25_RS05680↻	VlpF	34770
AXH25_RS05510↻		775
AXH25_RS05650↻	VlpB	592
AXH25_RS05670↻	VlpE	530
AXH25_RS05495↻		468
AXH25_RS05265↻		453
AXH25_RS05490↻		423
AXH25_RS05500↻		418
AXH25_RS05370↻	VlpH	231
AXH25_RS05655↻	VlpC	227
AXH25_RS05270↻		209
AXH25_RS05285↻	VlpL	192
AXH25_RS05355↻	VlpG	29
AXH25_RS05250↻		17
AXH25_RS05280↻		2
AXH25_RS05475↻		2
AXH25_RS05255↻		1
AXH25_RS05260↻		1
AXH25_RS05375↻		1
AXH25_RS05660↻		1
AXH25_RS05085↻		0
AXH25_RS05190↻		0
AXH25_RS05290↻		0
AXH25_RS05350↻		0
AXH25_RS05395↻		0
AXH25_RS05480↻	VlpJ	0
AXH25_RS05485↻		0
AXH25_RS05505↻		0
AXH25_RS05685↻		0
AXH25_RS05485↻		0
AXH25_RS05645↻	**VlpA**	0

Supplemental Table 1. PacBio reads for each strain CT13-2396 Vlp gene locus as determined from mouse reisolates from Experiments 1 -4. VlpA in bold denotes 0 reads for each reisolate except for Mouse 3 6dpi with 1 read.

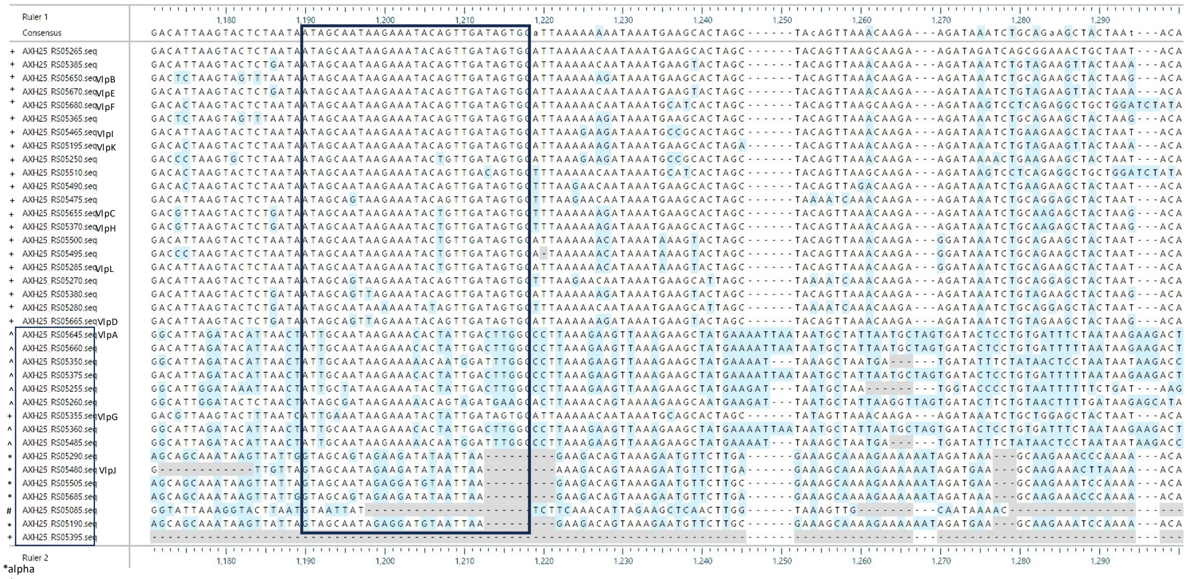

*alpha
# beta
+ delta
^ gamma

Supplemental Fig. 1. Vlp25R primer alignment with strain CT13 -2396 Vlp genes. Vlp25R primer region is boxed with nucleotide mismatches shaded. Legend indicates *vlp* subfamily for each gene. Area denoted is 5 ´ to 3´. Subfamily gamma, alpha, and beta with >2 mismatches are boxed.

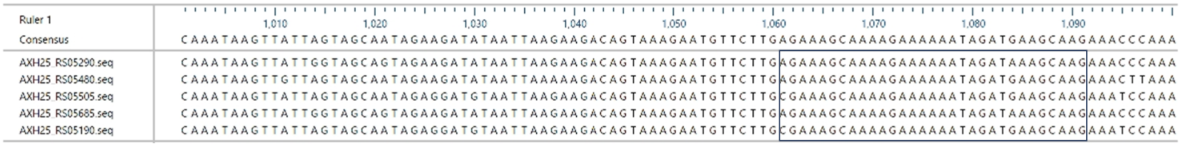

Supplemental Fig. 2. Vlp4R primer alignment with strain CT13-2396 alpha subfamily Vlp genes. Vlp4R primer region is boxed. Area denoted is 5′ to 3′.
